# Altering the Properties of Laccases from *Ensifer meliloti* (*Sinorhizobium meliloti*) and *Cerrena unicolor* by Chemical Modifications of Proteins

**DOI:** 10.3390/biom15040531

**Published:** 2025-04-04

**Authors:** Anna Pawlik, Radosław Drozd, Grzegorz Janusz

**Affiliations:** 1 Department of Biochemistry and Biotechnology, Institute of Biological Sciences, Maria Curie-Sklodowska University, Akademicka 19 St., 20-033 Lublin, Poland; grzegorz.janusz2@mail.umcs.pl; 2 Department of Microbiology and Biotechnology, Faculty of Biotechnology and Animal Husbandry, West Pomeranian University of Technology in Szczecin, 45 Piastow Avenue, 71-311 Szczecin, Poland; rdrozd@zut.edu.pl

**Keywords:** laccase, *Cerrena unicolor*, *Ensifer meliloti*, *Sinorhizobium meliloti*, chemical modification, thermostability

## Abstract

Due to their catalytic performance, laccases constitute one of the most promising groups of enzymes for potential applications in modern biotechnology. In this study, we aimed to chemically modify *Ensifer meliloti* (*Sinorhizobium meliloti*) and *Cerrena unicolor* laccase and comparatively characterize the structures of both enzymes. The most characteristic feature was the spatial localization of lysine residues, predominantly positioned distal to the active site region for both compared enzymes. The solvent-accessible surface area (SASA) analysis showed that bacterial laccase was characterized by a larger hydrophobic SASA than the fungal enzyme. The pK_a_ prediction identified only one Lys in the *E. meliloti* laccase structure susceptible to modification. Modifications were achieved by using mono- and bifunctional crosslinking agents, and glycosylations were also performed. The degree of protein modification ranged from 0% for glucose- and galactose-modified *E. meliloti* laccase and citraconic anhydride-modified (CA) *C. unicolor* laccase to 62.94% for the palmitic acid N-hydroxysuccinimide ester-modified *E. meliloti* enzyme. The stability of covalently modified laccases over a wide pH and temperature ranges and in the presence of inhibitors was investigated. Protein modifications with polymeric sucrose (PS) and ethylene glycol bis-(succinimidyl succinate) (EGNHS) significantly increased the activity of the bacterial and fungal laccases by 15 and 19%, respectively. Although pH optima remained relatively unchanged by modifications, certain variants, especially CA-modified bacterial protein and EGNHS-modified *C. unicolor* enzyme, exhibited improved stability at near-neutral pH (6–7). Modification of the bacterial enzyme with glutaraldehyde-carbodiimide (GA-CDI-ver) and of the fungal enzyme with CA was the most effective in improving its thermal stability. Chemical modifications using GA, CDI, GA-CDI, and PS allowed *E. meliloti* L 3.8 laccase to retain full activity in the presence of 5 mM NaI, whereas CA-, PS-, and EGNHS-modified *C. unicolor* variants retained their activity even at elevated NaCl concentrations. The results clearly demonstrate that the outcome of chemical modifications is closely linked to enzyme-specific structural features and that selecting an appropriate modification strategy is critical to achieving the desired effect.

## 1. Introduction

Most of the modern industry processes require biocatalysis based on enzymes with a stable structure in a wide range of physical and chemical factors influencing their activity, such as temperature, pH, and ion strength. Therefore, the design of modified enzymes may become highly important for biotechnological and pharmaceutical applications, e.g., the design of more effective or new catalysts [1,2,3].

Among many enzymes modified in various ways, laccases (EC 1.10.3.2) attract the attention of scientists from all over the world because they constitute one of the most promising classes of enzymes for future use in various fields. Genes encoding multicopper oxidase are found in nearly all kingdoms [4]. These enzymes may oxidize a broad range of organic substrates, including phenols, polyphenols, anilines, and even certain inorganic compounds, by a one-electron transfer mechanism [5,6,7,8,9,10]. The so-called broad-range substrate specificity displayed by laccases turns these enzymes into powerful tools already used in many areas of human activity: forest or textile industry [11,12,13], food production [14], cosmetics [15], medicine [16,17], and environmental protection [18,19].

To date, fungal and bacterial laccases have been most intensively studied, albeit for different reasons. The biological functions of laccases and laccase-like multicopper oxidases (LMCOs) are diverse and mainly include the degradation of lignin in the case of fungal enzymes. In the case of prokaryotic cells, they are related to morphogenesis and resistance to various stresses [4]. Extracellular fungal laccases are produced in higher amounts than bacterial laccases but display lower activities at higher temperatures, pH, or chloride concentrations [20]. The possible areas of laccase applications have been reported in many papers on enzyme production and purification, structure, genetics, and expression and finally on its chemical and engineered modifications [21,22,23,24,25,26,27]. However, relatively few chemically modified laccases are known. Covalent chemical modifications, crosslinking of enzyme crystals, modifications of enzyme surface amino groups, and incorporation of cofactors into protein molecules with original methods available for altering protein properties has now re-emerged as a powerful complementary approach to site-directed mutagenesis and directed evolution for tailoring proteins and enzymes [28,29,30]. *Trametes versicolor* or *Trametes hirsuta* laccase crosslinking resulted in an over four-fold increase in thermostability corresponding to its native form [1,31], whereas this enzyme stabilized through the formation of a surrounding polymeric network made of chitosan and 3-aminopropyltriethoxysilane showed more than 30-fold higher thermoresistance [32]. The same enzyme may be used for ferulic acid-mediated crosslinking of other proteins (*β*-lactoglobulin and α-casein) followed by changes in surface tensions in the protein [33]. Hydrophobized and hydrophilized laccase from *Cerrena unicolor* showed enhanced catalytic activity and higher pH and temperature stability in comparison to its native form [34].

Therefore, in the present study, we investigated the stability of chemically modified laccases over wide pH and temperature ranges and in the presence of inhibitors as factors limiting the industrial applications of these enzymes to select the most promising modifications. Next, we aimed to comparatively characterize the predicted structure of fungal and bacterial laccases to identify potential modification sites and elucidate the efficacy of the proposed chemical modifications.

## 2. Materials and Methods

### 2.1. Medium, Growth Conditions, and Preparation of Enzymes

*C. unicolor* C-139 was obtained from the culture collection of the Regensberg University. The fungus was maintained in 2% (*w*/*v*) malt agar slants. As an inoculum, pieces of agar overgrown with mycelia were incubated in the Lindenberg and Holm (LH) medium [35] at 28 °C for 7 days. The mycelial mats were subsequently collected and homogenized in a Waring blender. After inoculation with 2.5% (*v*/*v*) fungal biomass suspension, the mycelium was cultivated at 26 °C in 100 mL Erlenmeyer flasks in an incubator shaker (180 rpm).

The fermentor scale cultivation was performed in a 2.5 L Bioflo III fermentor (New Brunswick Scientific, New Brunswick, NJ, USA) containing 2 L of sterilized LH medium optimized as in [36] at 28 °C for 7 days. The fermentor was inoculated with fungal biomass suspension (10% of total volume), aerated at 1 L air per minute, and stirred at 100 rpm. Antifoam B emulsion (Sigma, St. Louis, MO, USA) was occasionally added to the fermentor cultures to break the foam. An amount of 10 mL of the culture was sampled every 24 h.

The post-culture liquid was centrifuged at 10,000× *g* on a K6 15 centrifuge (Sigma, Osterode am Harz, Germany) for 15 min. The supernatant was concentrated 10 times on the ultrafiltration system Pellicon 2 Mini holder (Millipore, Bedford, MA, USA) with an Ultracel mini cartridge (10 kDa cutoff) and used as the source of crude enzyme.

Chromatography was performed using a chromatographic EconoSystem (Bio-Rad, Richmond, VA, USA). The enzyme solution was loaded on a DEAE-Sepharose column (2.5 × 15 cm) pre-equilibrated with 20 mM Tris-HCL buffer (pH 6.5) with 0.1 M NaCl, and proteins were eluted with a 0.1–0.5 M linear gradient of NaCl at a flow rate of 1 mL/min.

*E. meliloti* L3.8 was obtained from the culture collections of the Department of Genetics and Microbiology, Maria Curie-Sklodowska University (UMCS) in Lublin, Poland. The stock culture of the bacterial strain was maintained on 79CA [37] agar medium at 30 °C for 2–4 days, and then used for seed culture inoculation. Next, *E. meliloti* was cultivated in 4 mL of liquid TY-Cu^2+^ medium as described by [38] at 30 °C for 3 days at 120 rpm. Liquid TY-Cu^2+^ medium (tryptone 5 g/L, yeast extract 3 g/L, CaCl_2_ × H_2_O 0.65 g/L, CuSO_4_ × 5 H_2_O 40 mg/L) was inoculated with a suspension containing 10^6^ cells/mL. Cultures were grown in 100 mL Erlenmayer flasks with 30 mL of medium in an incubator shaker (120 rpm) at 30 °C for 7 days.

*E. meliloti* cells were harvested by centrifugation (10,000× *g*, 15 min) and subjected to the laccase extraction procedure. Crude periplasmic laccase was obtained according to the protocol proposed by [39] and modified by [40]. Pelleted cells were suspended in 30 mM Tris–HCl buffer, pH 8.0, supplemented with 20% (*w*/*v*) sucrose, and treated with lysozyme (final concentration of 0.5 mg/mL) for 15 min at room temperature (RT). Next, a solution of EDTA, pH 8.0, (final concentration of 100 mM) was added and incubated for 20 min at RT. The homogenate was centrifuged (15,000× *g*, 30 min at 4 °C) and the clear supernatant was used as a crude enzyme source.

The crude enzyme extract was concentrated on the ultrafiltration system Amicon 8200 (Millipore, Bedford, MA, USA) with the YM-10 membrane (10 kDa cut off). The concentrated protein was loaded onto a DEAE-Sepharose (fast flow) chromatography column (1.5 × 25 cm) equilibrated with 50 mM Tris-HCl buffer (pH 7.2) and connected to the chromatographic system ÄKTAprime Plus (GE Healthcare, Uppsala, Sweden). The column was subsequently washed with buffer for 60 min, eluted with the same buffer supplemented with 0.5 M NaCl (linear salt gradient 0–0.5 M) at a rate of 1 mL/min, and 5 mL fractions were collected. The protein elution profile (at 280 nm) and laccase activity were recorded in each fraction. Fractions showing laccase activity were pooled, and the partially purified enzyme solution was used for further experiments.

### 2.2. Chemical Modifications of Laccases

For chemical modification of the laccases, typical initial values for each modification procedure were used: specific activity 20 U/mg and 2 mg/mL of protein. The description of the modifications is shown in Table A1. Appropriate control experiments (laccase without the modifying agent) were performed for each modification procedure to avoid any interference from the reaction mixture and compensate for any losses/increases in enzyme activity due to the procedure used. The modified enzymes were characterized by protein quantification using the BCA method (bicinchoninic acid assay), TNBS (2,4,6-trinitrobenzene sulfonic acid) [41], and standard activity assays [42] and subjected to further experiments. All measurements were performed in triplicates.

Protein concentration was determined using the BCA assay kit (Thermo Fisher Scientific, Rockford, IL, USA) by mixing 200 µL of working reagent (50:1, Reagent A:B) with 25 µL of sample. After incubation (37 °C for 30 min), spectrophotometric measurements were performed at 562 nm. Samples and blanks were included. BSA (bovine serum albumin) was used as a standard. All measurements were performed in triplicates.

The degree of modification (DM) was determined by the procedure based on the interaction of 2,4,6-trinitrobenzenesulfonic acid (TNBS) with the non-modified lysine residues of the enzyme [34,41]. In brief, 0.5 mL of a mixture of 4% NaHCO_3_, pH 8.5, and 0.1% TNBS (1:1 *v*/*v*) were added to 0.25 mL of a protein solution. The reaction mixture was incubated in the dark at 40 °C for 2 h. Next, 10% SDS (0.5 mL) and 1 M HCl (0.5 mL) solutions were added into each tube to terminate the reactions and absorbance of all samples at 335 nm was measured (Infinite 200 Pro microplate reader, Tecan, Crailsheim, Germany) against a blank containing water instead of the enzymes tested. The blank was prepared in the same way as the samples and controls. The number of free amino groups was evaluated using a standard curve prepared for lysine concentration (mM). The degree of modification was calculated according to Formula (1):

(1) $$DM\;\left[\%\right]=100-(\frac{CLys\_s}{CLys\_c})\times 100$$

where *C_Lys_s_* is the concentration (mM) of lysine residues per mg of enzyme preparation after modification and *C_Lys_c_* is the concentration (mM) of lysine residues per mg of an unmodified (control) enzyme preparation. All measurements were performed in triplicates.

Laccase activity was measured spectrophotometrically at 525 nm with 0.9 mL of 25 µM syringaldazine (4-hydroxy, 3,5-dimetoxybenzaldehyde) (Aldrich, St. Louis, MO, USA) as a substrate, suspended in 100 mM citrate-phosphate buffer, pH 5.5, and 0.1 mL of appropriately diluted enzyme preparation [42]. Enzyme and substrate blanks were included. One unit of laccase activity was defined as the amount of the enzyme catalyzing the production of one nanomole of colored product (quinone, εM = 65,000 M^−1^ cm^−1^) per second at 25 °C and pH 5.5. The activity was expressed as nkat/L. All measurements were performed in triplicates.

#### 2.2.1. EGNHS (Ethylene Glycol Bis-(Succinimidyl Succinate))

Crosslinking with EGNHS was performed as in Forde et al. [43] with modifications described below. EGNHS dissolved in 200 µL dimethyl sulfoxide (final concentration 5% *v*/*v*) was added slowly dropwise to 1 mL of laccase prepared in 100 mM potassium phosphate buffer, pH 7.4, and mixed (250 rpm) at RT. The reaction was terminated after 30 min by passage through a desalting column (70 × 15 mm, Pharmacia, Uppsala, Sweden) filled with Sephadex G-25 [34]. As a control in the EGNHS modification experiment, the enzyme was mixed with dimethyl sulfoxide and potassium phosphate buffer, pH 7.4, separately in the same ratios and treated in a similar manner to eliminate any contribution from the physical presence of these components.

#### 2.2.2. CA (Citraconic Anhydride)

The modification of the proteins using CA was performed according to the protocol developed by Forde et al. [43] with some modifications. CA (final concentration: 20 mM) was added to each 1 mL of the enzyme preparation in 0.1 M borate buffer, pH 9.0. The reaction was run at RT for 30 min, 250 rpm and terminated by passage through a desalting column (70 × 15 mm, Pharmacia, Uppsala, Sweden) filled with Sephadex G-25. As a control in the CA modification experiment, the enzyme was mixed with 0.1 M borate buffer, pH 9.0, separately in the same ratios and treated in a similar manner to eliminate any contribution from the physical presence of buffer components.

#### 2.2.3. GA (Glutaraldehyde) and CDI (Carbodiimide)

The modification procedures using GA, CDI, and GA-CDI were performed as described by Kucharzyk et al. [34]. In order to temporarily block the active site of the enzyme, protein samples were preincubated with 1 mM veratric acid (ver; 3,4-dimethoxybenzoic acid) in a volume ratio of 1:1 for 30 min at 25 °C. The modification procedure with 0.25 M GA, 0.25 M CDI, and a mixture of 0.125 M GA and 0.125 M CDI (GA-CDI) was then applied as previously mentioned in [34]. Briefly, 50 µL of modifying agent(s) and 0.7 mL of 100 mM citrate-phosphate buffer, pH 5.5, were added to 0.25 mL of preincubated with veratric acid enzyme solution or diluted enzyme solution. All mixtures were then incubated at RT with shaking (250 rpm). The reactions were terminated after 24 h by addition of 10 µL of NaBH_4_ (5 mg/mL), and then, the laccases were purified from non-reacted glutaraldehyde, carbodiimide, and a glutaraldehyde-carbodiimide mixture by passage through a desalting column (70 × 15 mm, Pharmacia, Uppsala, Sweden) filled with Sephadex G-25. As a control in the GA, CDI, GA-CDI modification experiment, the enzyme was mixed with 100 mM citrate-phosphate buffer, pH 5.5, separately in the same ratios and treated in a similar manner to eliminate any contribution from the physical presence of these components.

#### 2.2.4. N-HSP (Palmitic Acid N-Hydroxysuccinimide Ester)

The hydrophobic laccase was prepared as described in Kucharzyk et al. [34]. The N-HSP stock solution was prepared by dissolving the required amount of N-HSP in acetone (107 mg/mL). In order to temporarily block the active site of the enzyme, protein samples were preincubated with 1 mM veratric acid (ver) in a volume ratio of 1:1 for 30 min at 25 °C. The modification procedure using N-HSP was then applied as previously mentioned in [34]. Briefly, 0.75 mL of 0.2 M phosphate buffer, pH 8, containing 0.2% sodium taurodeoxycholate hydrate was added to 0.25 mL of preincubated veratric acid enzyme solution or diluted enzyme solution. Then, 0.5 mL of N-HSP solution was added (in portions, 0.05 mL every hour). The mixture was incubated at 4 °C with shaking (250 rpm) for 10 h. As a control in the N-HSP modification experiment, the enzyme was mixed with 0.2 M phosphate buffer, pH 8, containing 0.2% sodium taurodeoxycholate hydrate and acetone in the same ratios and treated in a similar manner to eliminate any contribution from the physical presence of these components.

#### 2.2.5. Mono and Disaccharides (Glc—Glucose, Gal—Galactose, Cel—Cellobiose, Lac—Lactose)

For modification experiments, glucose, galactose, cellobiose, and lactose were applied following the protocol developed by Kucharzyk et al. [34]. In order to temporarily block the active site of the enzyme, protein samples were also preincubated with 1 mM veratric acid (ver) in a volume ratio of 1:1 for 30 min at 25 °C. The modification procedure using Glc, Gal, Cel, and Lac was then applied as previously mentioned in [34]. Briefly, 50 µL of modifying agent and 0.7 mL of 100 mM citrate-phosphate buffer, pH 5.5, were added to 0.25 mL of preincubated with veratric acid enzyme solution or diluted enzyme solution. All mixtures were then incubated at RT with shaking (250 rpm). The reactions were terminated after 24 h by addition of 10 µL of NaBH_4_ (5 mg/mL), and then the laccases were purified from non-reacted carbohydrates by passage through a desalting column (70 × 15 mm, Pharmacia, Uppsala, Sweden) filled with Sephadex G-25. As a control in the Glc, Gal, Cal, and Lac modification experiment, the enzyme was mixed with 100 mM citrate-phosphate buffer, pH 5.5, in the same ratios and treated in a similar manner to eliminate any contribution from the physical presence of these components.

#### 2.2.6. PS (Polymeric Sucrose)

Modified enzymes were prepared according to Sundaram and Venkatesh [44] with some modifications. Oxidation of the polymer (Ficoll 400) was performed at 4.7 pH with 5-fold molar excess of sodium metaperiodate for 2 h at 25 °C with constant stirring in the dark. Unreacted periodate was removed by desalting using ultrafiltration system Amicon 8200 (Millipore, Bedford, MA, USA) with the YM-10 membrane (10 kDa cut off). The enzyme was covalently conjugated with oxidized Ficoll in molar ratios of 1:0.5. The protein–polymer mixture in 50 mM borate buffer, pH 8.0, was shaken for 2 h at RT and left overnight at 4 °C. Next, the coupling reaction was arrested with NaBH_4_ (5 mg/mL). As a control in the PS modification experiment, the enzyme was mixed with unoxidized Ficoll separately in the same ratios and treated in a similar manner to eliminate any contribution from the physical presence of carbohydrate polymers.

Modifications of *C. unicolor* C-139 laccase using glutaraldehyde, carbodiimide, palmitic acid N-hydroxysuccinimide ester, and mono- and disaccharides were already performed and the resulting proteins were initially characterized [34]. Therefore, in this work, the above-mentioned modifications were performed only for *E. meliloti* L3.8 laccase. The experiment was performed in three replicates.

### 2.3. Characterization of Modified Laccases

#### 2.3.1. Optimum pH Ranges and pH-Stability of Modified Laccases

The pH ranges for the native and modified laccase samples were measured using 0.1 M McIlvaine buffer with the pH range of 4.5–8.0. Laccase activity was measured using the Leonowicz and Grzywnowicz [42] method at pH 5.5. Specific activity was expressed as units of activity per mg of protein.

The pH stability of the modified enzymes was determined at 30 °C by preincubation in variable pH ranges (pH 4.0–8.0 McIlvaine buffer) at 4 °C followed by standard measurement [42] of residual activities in a time range of 0–30 days.

#### 2.3.2. Optimum Temperature and Thermal Stability of Modified Laccases

The optimum temperature ranges for the native and modified laccase forms were determined using a standard reaction mixture containing 0.5 mL of 0.1 M McIlvaine buffer (pH 5.5), 0.35 mL of deionized water, and 0.1 mL of the laccase sample. The samples were preincubated at temperatures ranging from 4 to 90 °C. Laccase activity was measured using the method proposed in [42] at pH 5.5.

The thermal stability evaluation was performed in buffer at different temperatures (30–90 °C). Aliquots were drawn in time intervals (5 min–24 h), and their residual enzyme activity was measured.

Thermal inactivation was assessed using a first-order activity decay model, and a semi-log plot of residual activity was generated based on the incubation time. The enzymatic activity decay was modeled by a first-order reaction: ln[A] = kdt + ln[A_0_], where A_0_ and A represent the initial and remaining enzyme activities at various time intervals. Here, kd (min^−1^) is the rate constant for enzyme inactivation, and t is the incubation time. The time (min) required to reach half of the initial enzyme activity (half-life, t_1/2_) was determined as ln (2)/kd.

#### 2.3.3. Laccase Inhibitors

The effect of selected chemical agents on laccase activity was measured by adding inhibitors of electron flow (halides: F-, Cl-, and I-), a reducing agent (dithiotreitol-DTT), and an ionic detergent (SDS) to the assay mixture at different concentrations (0.1–100 mM). Laccase activity was determined after adding the substrate according to a standard protocol [42].

### 2.4. Prediction of Ensifer Meliloti L3.8 and Cerrena Unicolor C-139 Laccase Structure

The amino acid sequences of the analyzed enzymes were retrieved from the GenBank database with the accession numbers AJT55525 (Lacc) for *E. meliloti* L3.8 (formerly known as *Sinorhizobium meliloti* L3.8) laccase and AEQ35306 (Lac1) for *C. unicolor* C-139 laccase. The signal peptide cleavage site was predicted using the SIGNALP 5.0 server. Amino acid sequences without the signal peptide were subsequently used for 3D structure prediction. The prediction of the 3D structure of the bacterial laccase was performed via the Robetta server using a fast and accurate deep learning-based method RoseTTAFold [45]. The best protein model, based on the GDT without further refinement, was chosen for subsequent analysis [46,47]. The quality of the protein models was evaluated by 3D Verify and Procheck via the SAVES server (https://saves.mbi.ucla.edu, accessed on 1 October 2024 ) [48,49]. The lysine residues’ SASA was calculated with the use of POPScomp (http://popscomp.org:3838/, accessed on 2 December 2024) at atomistic resolution with a 1.4 Å solvent radius (Table S2 and Table S3) [50]. The prediction of the reactivity of lysine residue based on the Henderson–Hasselbalch equation (*LysRe*) was calculated according to Formula (2):

(2) $${LysRe=10}^{\left(pH-pKa\right)}$$

where pH is the pH value used during modification protocol and pK_a_ is a pK_a_ value of lysine residues predicted with PROPKA 3.5.1 via APBS biomolecular solvation software suite (https://server.poissonboltzmann.org/pdb2pqr, accessed on 15 October 2024) [51]. The lysine residues with *LysRe* value below 0.1 were considered as low reactive, residues with values between 0.1 and 1.0 were considered as “semi-reactive”, and those above 1.0 as highly reactive.

## 3. Results

### 3.1. Influence of Modification on the Activity and Stability of E. Meliloti L3.8 and C. Unicolor C-139 Laccases

In this study, partially purified laccases from *E. meliloti* L3.8 and *C. unicolor* C-139 were subjected to chemical modifications aimed at changing the properties of the enzymes as a promising strategy for designing new biocatalysts in industrial applications. Free amino acid residues accessible for interaction were modified using (i) monofunctional citraconic anhydride (CA) and (ii) bifunctional crosslinking agents: palmitic acid N-hydroxysuccinimide ester (N-HSP), glutaraldehyde (GA), carbodiimide (CDI), and ethylene glycol bis-(succinimidyl succinate) (EGNHS). Additional glycosylations were performed with (iii) mono- and disaccharides: glucose (Glc), galactose (Gal), cellobiose (Cel), lactose (Lac), and polymeric sucrose (PS). Modifications using N-HSP, GA, CDI, Glc, Gal, Cel, and Lac were performed only for *E. meliloti* L3.8 laccase. Equivalent modifications for *C. unicolor* C-139 laccase were already the subject of our previous work [34]. The modifications introduced to the proteins are briefly listed in Table A1. Modified laccases were analyzed comprising basic enzyme characteristics, i.e., pH and temperature optima, pH and thermal stability, and inhibitory resistance. Moreover, the level of activity of both enzymes, compared to the control experiments, was determined in order to estimate which of the modification strategy allowed retaining maximum laccase activity. The degree of protein modification (DM) was also evaluated by spectrophotometric determination of the amount of covalently modified amino acids (lysines) on the protein surface (Table 1). It appeared that only the modification of lysine residues using N-HSP drastically lowered enzyme activity to 40% (at the same time, the DM was the highest and amounted to 59–62%), whereas the other methods allowed retaining at least 77% of laccase residual activity. Interestingly, the protein modifications with PS and EGNHS significantly boosted the activity of the bacterial and fungal laccases by 15 and 19%, respectively. In general, the proposed chemical modifications had no significant impact on the pH optimum of the analyzed laccases. A slight pH optimum shift (c.a. 0.5 pH unit), compared to the control experiments, was noticed except for glycosylated bacterial laccase (Glc-EM and PS-EM), where the value reached pH 7. In contrast, no increase in the temperature optimum for both modified enzymes was observed. In turn, a few modified *E. meliloti* L3.8 laccases appeared to have a lowered temperature optimum, compared to the control experiment (Table 1).

**Figure 1:**
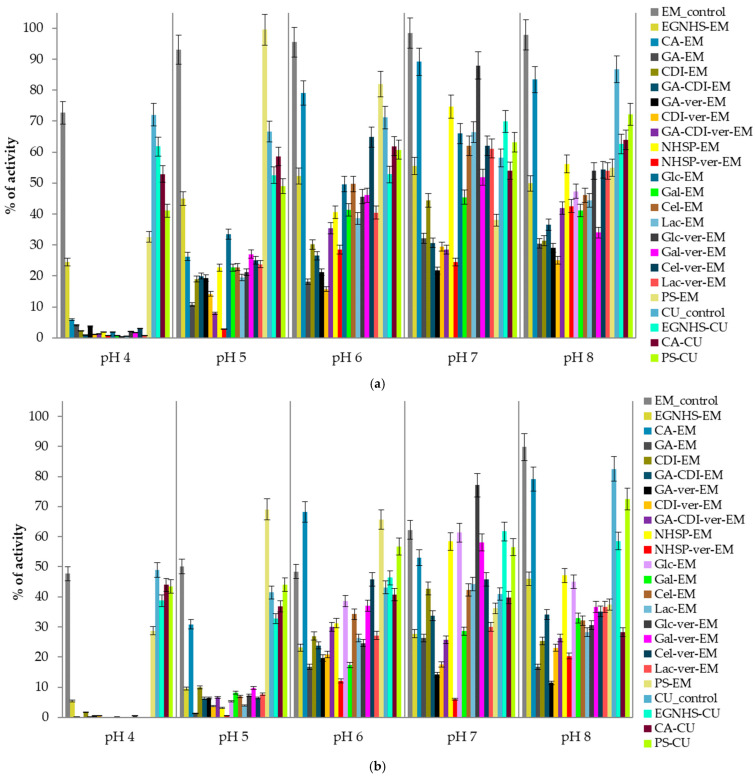
pH stability of *E. meliloti* L3.8 and *C. unicolor* C-139 laccases measured at the 15th (**a**) and 30th days (**b**) of the experiment; data are mean ± SD for three measurements (n = 3); the activity measured for each modified enzyme on day zero is considered to be 100% activity.

The pH stability of the modified enzymes was analyzed in variable pH ranges (pH 4.0–8.0) after 1 h, 2 h, 3 h, 6 h, 12 h, 24 h, 7 days, 15 days, 20 days, and 30 days of preincubation at 4 °C. Due to the high number of results and since the data from the 15th and 30th days were considered as the most significant and discriminatory, only these results are presented (Figure 1). In general, the glycosylation of both laccases with polymeric sucrose (PS) increased their pH stability (approx. 3–20%) in pH 5–7. The modification of the *E. meliloti* L3.8 laccase with monofunctional citraconic anhydride (CA) notably improved the stability by approx. 20% at neutral pH, whereas the EGNHS crosslinking of the *C. unicolor* C-139 enzyme resulted in boosting laccase activity by 3% and over 21% at pH 6 and 7, respectively, in comparison to the native enzymes (Figure 1b). The thermal stability of the modified proteins was analyzed in a temperature range from 30 °C to 90 °C, every 10 °C in time intervals (5, 15, 30, 60, 120, 180, 360, 720, and 1440 min). For both enzymes, 60 °C was chosen as a critical temperature for measuring thermal inactivation due to the significant vulnerability of all the modified and unmodified proteins and the observable discrepancies in the assumed time of the experiment (Table 2). The obtained results showed that the citraconic anhydride-modified *C. unicolor* C-139 laccase was characterized by higher thermal stability at 60 °C than its bacterial counterpart, as reflected by R values of 37.5 for CA-CU and 0.3 for CA-EM, respectively (Table 2). The modification of the *E. meliloti* L3.8 laccase by EGNHS, CDI, GA-CDI, and GDA-CDI-ver significantly improved the enzyme’s thermal stability. The highest improvement was observed for GA-CDI-ver-EM, where an over four-fold increase in the half-life time, compared to the non-modified form of the enzyme, was observed. The modification using PS was also beneficial for bacterial laccase thermal stability, extending the half-life time two-fold. The other modifications did not influence the enzyme thermal stability significantly or even led to a substantial reduction in the half-life time. In this case, the highest negative effect of the modification on enzyme thermal stability was observed for NHSP- ver-EM, NHSP-EM, and CA-EM (Table 2).

In the last stage of the experiment, the activity of the modified enzymes against selected inhibitors, i.e., NaF, NaI, NaCl, SDS, and DTT, was examined (Table S1). NaF and NaI were chosen as typical electron transfer disruptors in laccase copper centers. NaCl is often used as a component of elution buffers during laccase purification, whereas SDS and DTT are denaturing agents commonly used in electrophoresis protocols. All the reagents were tested in the concentration range from 0 to 100 mM. DTT appeared to be the most effective activity inhibitor for both the control and modified enzymes. In the presence of 0.1 mM DTT, the laccase activity dropped to less than 1%. However, the effect of SDS appeared to be more diversified. In comparison to the native laccases, the monofunctional citraconic anhydride treatment led to a significant decrease in the activity of the bacterial enzyme (12.6%), while the treatment with bifunctional ethylene glycol bis-(succinimidyl succinate) resulted in a substantial increase in enzyme activity and stability in the presence of 1 mM SDS. In turn, it seems that some of the modifications improved the resistance of the laccases to halides. In general, the *C. unicolor* C-139 laccase treated with EGNHS appeared to be more stable in the presence of halides. The bacterial laccase crosslinked with GA appeared to be active (92%) even at 10 mM of NaCl. A similar effect was observed after the modification of the fungal enzyme. This laccase modified with CA, EGNHS, and PS exhibited higher activity in comparison to the unmodified protein even when the NaCl concentration increased up to 50 mM. Moreover, the active site blocking in the case of the bacterial laccase had no/minor effect on the results. It was found that the *E. meliloti* L3.8 laccase modified with GA, CDI, GA-CDI, or PS and the *C. unicolor* laccase crosslinked with EGNHS maintained their activity up to 100% at the 5 mM concentration of NaI, whereas the *C. unicolor* laccase treated with CA and EGNHS was slightly resistant to inactivation in the presence of up to 5 mM NaF.

### 3.2. Comparative Analysis of the Structural Properties of E. Meliloti L3.8 and C. Unicolor C-139 Laccase

**Figure 2:**
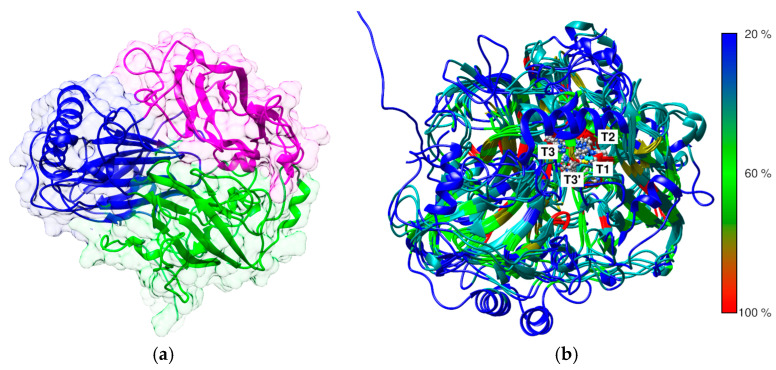
Predicted structure of *E. meliloti* L3.8 laccase. The recognized domain A is colored blue, domain B is colored purple, and domain C is colored dark green. The domain ranges were assigned according to SWORD2 partition algorithm analysis results [54] (**a**). The superimposition of the 3D structures of fungal and bacterial laccases from the resources of the Protein Data Bank and predicted structures of *E. meliloti* L3.8 and *C. unicolor* C-139 laccases with the highlighted probable localization of the three copper centers. Protein models are colored in rainbow scale to indicate the percent sequence similarity/identity calculated from multiple alignment (Figure 3) (**b**).

The determination of the three-dimensional (3D) structure of enzymes is fundamental to understanding the impact of structural modifications on their catalytic function and biochemical activity. Alterations, even at the level of a single amino acid residue, can lead to significant changes in substrate affinity, catalytic turnover rate, or enzyme stability, all of which are critical in determining the enzyme’s suitability for chemical modifications [52,53]. The laccase from *C. unicolor* C-139 showed high sequence and structure identity with several 3D structures of fungal laccases in the Protein Data Bank (PDB). Specifically, it shared 82.8% sequence identity with the laccase from *Cerrena* sp. RSD1 (PDB ID 5Z1X), 66.6% with *Trametes versicolor* (PDB ID 1GYC), and 65.6% with *Trametes trogii* (PDB ID 2HRG) (Figure S1). The laccase from *E. meliloti* L3.8 did not show close structural homology with fungal laccases and was instead more closely related to copper oxidases of bacterial origin, such as the CueO laccase from *Escherichia coli* (PDB ID 1N68) and the CotA laccase from *Bacillus subtilis* (PDB ID 1W6L). Meanwhile, structural comparison revealed that laccase from *E. meliloti* L3.8 showed 22.5% sequence similarity and an RMSD of 1.6 Å, compared with laccase from *C. unicolor* C-139 (Figure 2 and Figure S1).

**Figure 3:**
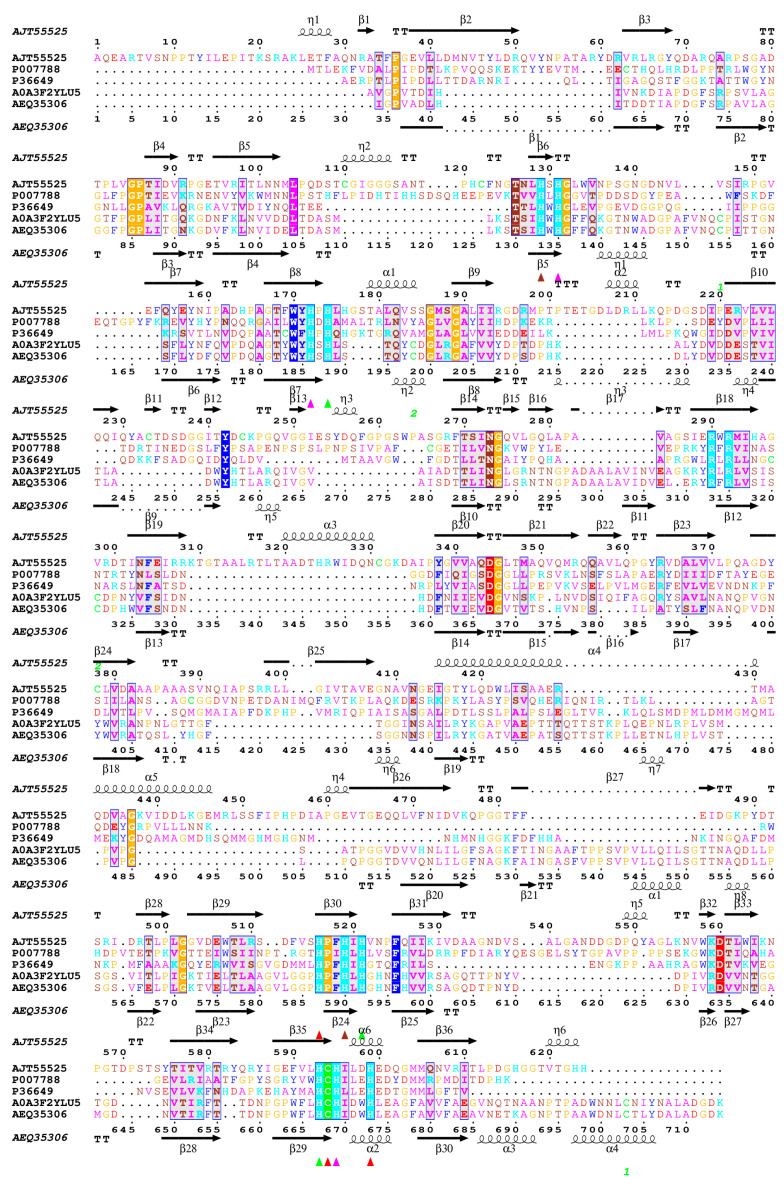
Structure superimposition and sequence-based multiple alignment of analyzed bacterial and fungal laccases with selected 3D structures available in the resources of the Protein Data Bank (PDB). The protein sequences were retrieved from GenBank under the following accession numbers: *E. meliloti* L3.8 (AJT55525), *B. subtills* CotA (P007788, PDB id 1W6L), *E. coli* (P36649, PDB id 1N68), *C. unicolor* C-139 (AEQ35306), and *Cerrena* sp. RSD1 (A0A3F2YLU5, PDB id 5z1x). The colored triangles indicate amino acid residues that coordinate copper-atoms T1 (▲), T2 (▲), T3 (▲), and T3′ (▲) in the laccase structure. The amino acid sequence multiple alignment was performed using Clustal Omega via UCSF-Chimera 1.18 [55] and visualized using ESPript 3.0 [56].

The predicted 3D structure of the bacterial laccase monomer exhibits the typical organization for small blue copper proteins and consists of three successively arranged domains with a fold similar to the β-barrel type architecture related to multicopper oxidase (PF00394) (Figure 2). The multiple alignment between known structures of bacterial and fungal laccases showed low sequence similarity between the selected laccases with the known 3D structure, confirming high divergence at the primary level of laccase structure organization (Figure 2). However, it revealed a high conservation of copper atom coordination/binding sites, and the localization of three probable copper centers was identified. The first mononuclear copper center (T1) localization was found in domain C (Figure 2 and Figure 3), comprising His 519, Cys 594, and His 599. The next copper center, called the tri-nuclear cluster (TNC, T2/T3), was localized at the interface between domains A and B (blue and purple, Figure 2). The TNC includes a mononuclear T2 copper center, probably coordinated by His 521, His 593, and His 174. The remaining copper centers are probably coordinated by His 131, His 172, and His 595 for T3 and His 129 and His 519 for T3′.

It was assumed that the performed modifications of the compared laccases were targeted primarily at lysine residues, which are often located on the solvent-accessible surface of proteins, making them particularly suitable for chemical or enzymatic modifications. Due to their positively charged side chains at physiological pH, lysines contribute to protein–protein interactions, stability, and function [57,58]. Their accessibility to the solvent is one of the obvious factor considered critical to their efficiency and likelihood of modification, and lysine residues highly exposed to the solvent are potentially more reactive, making them more likely to undergo modification [59].

**Figure 4:**
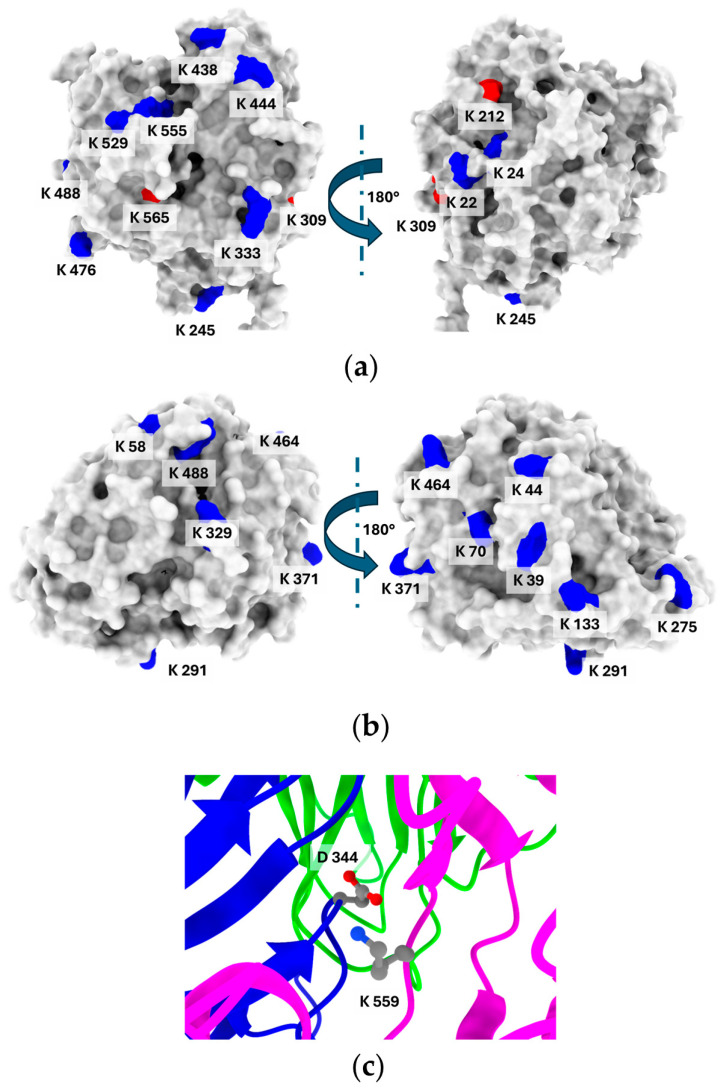
The distribution of lysine residues on the surface of laccase from *E. meliloti* L3.8 (**a**) and *C. unicolor* C-139 (**b**). Lysine residues marked in red are exposed to less than 20% of their overall solvent-accessible surface area (SASA). (**c**) Interdomain region of *E. meliloti* L3.8 laccase showing interactions of Lys 559 and Asp 344. The surface analysis was performed using POPS (Table S2 and Table S3) [50].

The number of lysine residues varies in the structure of the compared enzymes. The *E. meliloti* laccase contains fourteen lysine residues, from which, according to their SASA (solvent-accessible surface area) analysis, eight are significantly exposed to the solvent (<20% of SASA) and can be considered as possible modification sites (Figure 4). In turn, on the surface model of *C. unicolor* C-139 laccase, eight lysine residues out of eleven visible in the amino acid sequence were identified. The most characteristic feature was the spatial localization of the lysine residues, predominantly positioned distal to the active site region for both compared enzymes. This suggests that possible modification will not result in changing the local environment of the substrate binding site and will not influence the catalytic efficiency of the enzyme. The hydrophilicity/hydrophobicity surface analysis of the compared laccases revealed that the bacterial laccase had a hydrophobic solvent-accessible surface area (SASA) of 16,789.74 Å^2^, compared to 12,121.44 Å^2^ for the fungal laccase. However, the ratio of hydrophilic to hydrophobic surface ratio was similar for both enzymes and was 1.2 for *E. meliloti* L3.8 laccase and 1.34 for *C. unicolor* C-139 laccase (Table S4).

Lysine accessibility is indeed only one factor that indirectly influences its reactivity. When multiple lysine residues have similar solvent accessibility, this parameter alone cannot reliably indicate the most probable site for lysine-dependent modification. Additional factors, including the local microenvironment, electrostatic interactions, and hydrogen bonding, play key roles in modulating lysine reactivity [60]. These elements can significantly affect the likelihood of lysine engaging in nucleophilic interactions, especially under conditions where its protonated state would typically limit reactivity. This interplay suggests that ideal modification sites on lysine residues should exhibit a substantial downshift in pK_a_. Typically, lysine residues on protein surfaces have a pK_a_ of around 10.4, which causes them to be protonated and positively charged under modification conditions (pH 7.4–9.0), limiting their nucleophilicity. For lysine to participate effectively in nucleophilic interactions, a pK_a_ shift of about 2–3 units lower would be required [61]. The pK_a_ prediction revealed that only one lysine residue, Lys 559, in the *E. meliloti* L3.8 laccase structure showed a noticeable decrease in pK_a_ to 8.92. This residue participates in the formation of a salt bridge with Asp 344 and is additionally thoroughly buried in the interdomain region, which potentially affects its accessibility and reactivity (Figure 4c). The pK_a_ values for other lysine residues ranged from 10.05 (Lys 565) to 11.44 (Lys 476), indicating that, under the modification conditions, these residues likely exhibit low reactivity based on their *LysRe* values at the relevant pH (Table S2). Integration of previous analyses with information on salt bridge formation narrows down the likely modification sites to four solvent-accessible lysine residues—Lys 20, 476, 488, and 529. This is consistent with the results of the modification degree analysis for CDI, EGNHS, and GA (Table 1), indicating that up to four lysine residues can be modified. For *C. unicolor* C-139 laccase, the predicted pK_a_ values for lysine residues ranged from 10.31 (Lys 312, Lys 464) to 11.52 (Lys 58). Remarkably, no lysine residue with a significantly reduced pK_a_ was detected that could be unequivocally identified as the most probable modification site. In the 3D structure of *C. unicolor* C-139 laccase, out of eleven lysine residues, seven are involved in salt bridge formation. Excluding these, Lys 70, Lys 291, Lys 329, Lys 371, and Lys 464 can be considered potential candidates for modification.

## 4. Discussion

For industrial applications, there is a growing demand for enzymes with improved properties that exceed those of their natural counterparts. In addition to strategies involving genetic and protein engineering to tailor enzyme structures, numerous chemical modification techniques are used to refine and enhance the functional properties of enzymes. This strategy enables the covalent integration of chemical moieties into enzymes beyond the boundaries of translation and post-translational modifications. While chemical modifications increase the functionality of enzymes, their effects remain challenging to predict, as they depend on the structure of the enzyme and the properties of the introduced groups [3]. Among the wide range of industrial enzymes, laccases remain extensively investigated due to their ability to oxidize a wide variety of substrates, thus enabling diverse applications. However, their instability under harsh conditions is a major limitation, requiring the use of stabilization strategies such as chemical modification, immobilization or other techniques [43,62].

The predicted 3D structure of the *E. meliloti* L3.8laccase monomer exhibited a typical architecture related to class I multicopper oxidases (MCOs), mainly from fungi but also from some bacteria and insects [63]. As expected, sequence and structure comparisons revealed a high degree of conservation in the amino acid motifs responsible for Cu^2+^ coordination in bacterial and fungal laccases [64]. In contrast, the substrate-binding loop region displayed significant structural divergence, likely contributing to differences in substrate specificity and selectivity [65,66]. It is worth noting that low amino acid sequence homology in this region appears to be a distinguishing feature of *E. meliloti* L 3.8 laccase compared to *C. unicolor* C-139 laccase, which potentially influences its catalytic properties, substrate preferences, and the observed effects of applied modifications.

The efficiency or susceptibility to various modifications depends strongly on the properties of the laccase molecular surface, i.e., its hydrophobicity, polarity, and the occurrence of specific amino acids with amino or carboxyl groups, etc. [67]. This type of amino acid is usually the main target for the formation of a linkage between various modification agents [68]. The conducted modifications were mainly addressed to lysine residues, which differ not only in number in the analyzed enzymes but also in their location, which may determine their suitability for modification, as well as the possibility of the formation of intraprotein bridges between the lysine and the crosslinking agent (Figure 4) [69]. The potentially highly reactive amino groups also make lysine the most conventional and facile target for modification [70]. However, a detailed analysis showed that out of eleven residues, only five could be considered as the most likely candidates for modification in the case of *E. meliloti* L3.8 laccase. In the conducted study, a monofunctional citraconic anhydride (CA) and N-HSP were used, both of which are capable of reacting with a single amino group. Additionally, a larger set of bifunctional crosslinkers, CDI, GA, and EGNHS, was examined. In the case of N-HSP, lysine residues serve as the attachment sites for palmitic chains [71]. Among the bifunctional crosslinkers, glutaraldehyde (GA) reacts with proteins in a more complex manner, as it can engage in multiple interactions. The most likely mechanism involves the reaction between lysine residues (i.e., amino groups) of the protein and a polymeric form of glutaraldehyde [43,72]. In contrast, EGNHS can form an intramolecular crosslink between lysines, but can also react monofunctionally, modifying lysines without forming a crosslink [73]. Glycosylations of laccases performed with mono- and disaccharides, i.e., Glc, Gal, Cel, Lac, and PS, can also be targeted to amino groups [34,44]. The location of lysine residues, determined from the predicted 3D structures of the studied laccases, suggested that the introduced modifications would not alter the local environment of the substrate binding site and would not directly influence the catalytic efficiency of the enzyme. However, N-HSP modification drastically reduced the laccase activity of *E. meliloti* L3.8 to 40%, which correlated with the highest degree of modification. A similar effect was also observed for laccase from *C. unicolor* C-139 [34]. In contrast, PS modification led to a 19% increase in *E. meliloti* L3.8 laccase activity, while EGNHS treatment resulted in a 17% increase in *C. unicolor* C-139 laccase activity. Forde et al. [43] observed a slight increase in *Myceliophthora thermophila* laccase activity following EGNHS modification, whereas *Trametes hirsuta* laccase decreased. These findings highlight that structural differences among laccases influence the outcome of chemical modifications, which was also noted in this study. The observed activity is a function of various operational parameters influencing the modified enzyme, including pH, thermal catalytic efficiency, and stability optima [74].

The first operational parameter analyzed, the pH optimum of *E. meliloti* L3.8 laccase, changed significantly after glycosylation with glucose and polymeric sucrose, increasing by about 1.0 pH unit toward the neutral range. In contrast, most other modifications resulted in a more moderate shift of around 0.5 pH units. As reported by Giri et al. [3], enzyme modifications performed with dibasic anhydrides slightly shift pH optima to alkaline. Kucharzyk et al. [34] managed to change laccase pH optima towards acidic values by modifying its structure with sugar moieties but this was not observed after PS treatment. Similar observations were made by Danait-Nabar et al. [27] when *M. thermophila* laccase was modified with phthalic and 2-octenyl succinic anhydrides. In addition to its catalytic efficiency, this enzyme is considered suitable for industrial use and should be characterized by improved stability at various pH [75]. Although the various laccase modifications presented here did not alter its structure enough to observe significant shifts in the optimal pH, an improvement in stability at neutral pH was observed. The changes in pH catalytic optima and stability after glycosylation in *E. meliloti* L3.8 laccase are due to structural and biochemical modifications. Glycosylation alters the tertiary structure of the enzyme, affecting substrate binding and catalytic efficiency, and shifts the electrostatic environment, stabilizing key residues and protonation states at a higher pH [76,77]. It also enhances enzyme rigidity, reducing pH-induced denaturation, and modifies hydration dynamics, influencing proton availability and pK_a_ shifts [78,79]. Furthermore, glycosylation influences enzyme–substrate affinity and cofactor coordination, optimizing electron transfer and contributing to pH shift [80,81]. These factors likely collectively enhance enzyme stability and catalytic performance at a more neutral pH.

The second set of operational parameters of the compared laccases, such as temperature-depended catalytic optima and stability, were also modified, and the difference in the observed effect was a consequence of the structural discrepancy between the compared laccases. However, of all those tested, only modification by GA-CDI and N-HSP changed the temperature optimum of bacterial laccase, lowering it from 80 °C to 60 °C. A similar effect was observed for *M. thermophila* laccase modified with 2-octenyl succinic anhydrides where the thermal optima decreased from 60 °C to 20 °C [27], whereas Kucharzyk et al. [34] did not observe significant changes in the temperature optima of *C. unicolor* C-139 laccase after modification with chemicals with similar properties. Nwagu et al. [82] successfully shifted the optimal temperature of amylase by changing its structure by attaching heteropolysaccharides such as chitosan; however, no significant effect of the tested mono- and polysaccharides on the compared laccase was observed.

Our results (Table 2) indicate that fungal laccase is less resistant to elevated temperatures than bacterial laccase. Interestingly, the enzymes differ in their lysine content—bacterial laccase contains a larger number of lysine residues compared to its fungal counterpart. Increased lysine content increases the potential to form stabilizing electrostatic interactions, in particular salt bridges. The 3D structural models of both enzymes revealed that the potential number of salt bridges is approximately 10 for the bacterial laccase and 7 for the fungal laccase (Table S2 and Table S3). This expanded network of salt bridges in the bacterial enzyme may contribute to a more rigid and robust structure, which minimizes conformational fluctuations at elevated temperatures and increases its thermal stability [83,84]. These structural properties may partially explain the observed negative effect of CA and N-HSP modifications, which result in a drastic reduction of the thermal stability of *E. meliloti* L3.8 laccase, probably due to the destruction of salt bridges. Second, hydrophilicity/hydrophobicity surface analysis of the laccases revealed that the bacterial enzyme exhibited a slightly more hydrophobic SASA compared to the fungal counterpart. Further increase in hydrophobicity due to the addition of a significant number of palmitic acid molecules, observed in the highest degree of modification, may lead to reduced thermostability, mainly due to increased hydrophobic interactions between enzyme molecules, resulting in aggregation and structural destabilization at elevated temperatures. A particularly noteworthy modifier was polymeric sucrose (PS), which yielded an excellent balance between thermostabilization and activity preservation. In *E. meliloti* L3.8 laccase, PS treatment extended the thermal half-life 2-fold (from 219.7 min to 437.3 min), while increasing relative activity to 119.7%. Even more strikingly, in *C. unicolor* C-139 laccase, PS modification resulted in a more than 12-fold increase in half-life (from 47.7 min to 600.8 min), with 100.7% activity retained. These effects are primarily attributed to increased structural rigidity of laccases resulting in the formation of hydrogen bonds between the polymers and the protein surface (i.e., crosslinking), as well as the formation of a hydrophilic layer that shields the enzyme’s hydrophobic regions from water [3,85]. A similar effect can be attributed to the modification of the enzyme structure by CA, which increased the surface hydrophilicity and consequently thermostability. This positive effect was particularly pronounced for *C. unicolor* C-139 laccase, where thermostability increased 37-fold. However, the same modification had the opposite effect on *E. meliloti* L3.8 laccase, significantly reducing its stability. The effect of CA modification depends strongly on the structural properties of the enzyme. While CA-modified α-chymotrypsin and amylase exhibited enhanced thermostability, no significant effect was observed for lipase [86,87]. The second group of tested modifying agents, including GA and EGNHS, was able to form intraprotein crosslinks, which probably contributed to increased thermostability by enhancing enzyme rigidity [88]. Analysis of the distances between the Cα and εN atoms of lysine residues in the predicted 3D structures of both laccases confirmed the potential for intraprotein crosslink formation (Table S5 and Table S6 and Figure S2 and Figure S3). GA, in combination with CDI, improved the thermostability of *E. meliloti* laccase by 4.3-fold (t_1/2_ = 938.7 min), while retaining 95.8% of its native activity. Similarly, EGNHS treatment increased the stability of *C. unicolor* laccase by 2.3-fold (t_1/2_ = 111.1 min), with 115.6% activity retained. These results highlight both agents as particularly promising stabilizing modifiers, providing substantial enhancement of thermal resistance without severe impact on enzyme performance.

In addition to the factors described above, the broader application of modified enzymes may also depend on their stability in environments containing a variety of compounds that can potentially alter their activity, inhibit their function, or reduce their overall effectiveness in catalytic processes. PS- and GA-modified *E. meliloti* L3.8 laccase retained more than 90% of its original activity after exposure to NaI and NaCl at concentrations up to 10 mM. However, *C. unicolor* C-139 laccase modified with CA, PS, and EGNHS demonstrated increased resistance to NaCl at concentrations up to 50 mM. This stabilization can be attributed to the ability of polysucrose to form a protective layer around the enzyme, shielding it from denaturing agents and maintaining a stable hydration shell, which is crucial for maintain enzyme stability and activity [89]. Moreover, PS can modulate electrostatic interactions, reducing the disruptive effects of salts and further supporting the integrity of the enzyme’s structure [90]. All the laccases analyzed were found to be highly sensitive to DTT. This reducing compound is a typical inhibitor of laccases due to its ability to reduce the disulfide bridges present in the active center of the enzyme, which stabilize the protein structure and hold the copper ions in the correct configuration [91]. However, although some modifications provided beneficial effects, most had a neutral impact, contributing primarily to increased solubility rather than significant changes in stability with respect to the factors analyzed. Notably, modifications of *M. thermophila* laccase with CA made this enzyme more susceptible to EDTA, β-mercaptoethanol, and sodium azide, suggesting that certain modifications may also introduce vulnerabilities depending on the specific chemical environment [27]. Resistance to excessive salt concentrations may be a desired enzyme characteristics when considering wastewater removal [92]. Among all tested modifying agents, GA-CDI-ver, PS, and EGNHS emerged as the most effective in enhancing laccase thermal stability, while preserving or improving enzymatic activity. These modifiers represent promising candidates for future enzyme engineering and immobilization strategies.

## 5. Conclusions

The performed study clearly confirmed that efficient modification of enzyme properties is closely linked to structural characteristics, particularly the molecular surface. The predicted 3D structures of *E. meliloti* L3.8 and *C. unicolor* C-139 laccases exhibited similar folding patterns typical for multicopper oxidases but differed notably in surface architecture, lysine distribution, and residue accessibility. These structural differences, affecting the reactivity of surface-exposed residues, likely determined their distinct responses to chemical modification agents, resulting in varied effectiveness of individual modifiers between the enzymes. Interestingly, the modifications of the proteins with PS and EGNHS increased the activity of the bacterial and fungal laccases by as much as 15 and 19%, respectively. Although pH optima remained relatively unchanged by modifications, certain variants, especially PS-CU, PS-EM, CA-EM, and EGNHS-CU, exhibited improved stability at near-neutral pH (6–7). Enhanced thermal stability was observed in some cases, particularly following GA-CDI and CA treatments, though modified fungal laccase intrinsically maintained higher thermal resistance. Chemical modifications using GA, CDI, GA-CDI, and PS allowed *E. meliloti* L 3.8 laccase to retain full activity in the presence of 5 mM NaI, whereas CA-, PS-, and EGNHS-modified *C. unicolor* C-139 variants retained their activity even at elevated NaCl concentrations.

The findings of our study clearly demonstrate that the outcome of chemical modifications is closely linked to enzyme-specific structural features, and further highlight that the selection of an appropriate modification strategy and reagent is critical to achieving the desired effect. In the case of the laccases analyzed, we successfully identified a combination of modifiers that significantly improved the thermal stability of the enzyme without compromising its catalytic activity, which may be of potential use in future applications requiring increased resistance to elevated temperatures.

## Figures and Tables

**Table 1:** Residual activity, degree of protein modification, pH, and temperature optima of modified laccases; the measurements were performed in triplicates (n = 3).

Modification	Recovery of Initial Activity [%]	DM * [%]	pH Optimum	Temperature Optimum [°C]
EM_control	100	-	6.0	80
EGNHS-EM	82.0 ± 1.24	30.78 ± 0.8	6.5	80
CA-EM	90.1 ± 4.16	28.64 ± 1.13	6.5	80
GA-EM	97.5 ± 1.25	22.07 ± 0.86	6.5	80
CDI-EM	98.3 ± 5	13.76 ± 0.48	6.5	80
GA-CDI-EM	105.0 ± 0	5.53 ± 0.3	6.5	60
GA-ver-EM	84.2 ± 2.92	43.47 ± 1.34	6.5	80
CDI-ver-EM	104.2 ± 1.25	18.72 ± 0.63	6.5	80
GA-CDI-ver-EM	95.8 ± 1.25	33.08 ± 0.49	6.0	60
NHSP-EM	42.2 ± 2.32	59.18 ± 0.57	6.5	60
NHSP-ver-EM	40.7 ± 1.58	62.94 ± 0.77	6.0	80
Glc-EM	87.8 ± 0.46	0.00	7.0	80
Gal-EM	77.2 ± 5.36	3.48 ± 0.1	6.5	80
Cel-EM	88.3 ± 0.2	11.97 ± 0.3	6.0	80
Lac-EM	92.1 ± 4.44	3.81 ± 0.12	6.0	80
Glc-ver-EM	80.4 ± 2.85	0.00	6.5	80
Gal-ver-EM	85.6 ± 1.19	0.00	6.0	80
Cel-ver-EM	103.7 ± 0.46	0.97 ± 0.7	6.5	70
Lac-ver-EM	106.2 ± 3.05	8.33 ± 0.29	6.5	80
PS-EM	119.7 ± 2.3	0.69 ± 0.13	7.0	80
CU_control	100	-	5.5	80
EGNHS-CU	115.6 ± 0.91	57.01 ± 1.45	5.5	80
CA-CU	105.0 ± 1.63	0.00	6.0	80
PS-CU	100.7 ± 0.53	35.02 ± 0.69	5.5	80

* degree of protein modification.

**Table 2:** Thermostability of modified and native *E. meliloti* L3.8 and *C. unicolor* C-139 laccases during 180 min of incubation at 60 °C; the measurements were performed in triplicates (n = 3).

Modification	kd (min^−1^) *	t_1/2_ (Min) ^†^	R ^#^
EM_control	31.55 × 10^−4^	219.7	-
Glc-ver-EM	44.81 × 10^−4^	154.7	0.7
Glc-EM	53.36 × 10^−4^	129.9	0.6
Gal-ver-EM	27.45 × 10^−4^	252.5	1.1
Gal-EM	59.55 × 10^−4^	116.4	0.5
Cel-ver-EM	39.54 × 10^−4^	175.3	0.8
Cel-EM	48.10 × 10^−4^	144.1	0.7
Lac-ver-EM	38.90 × 10^−4^	178.2	0.8
Lac-EM	70.87 × 10^−4^	97.8	0.4
GA-ver-EM	45.27 × 10^−4^	153.1	0.7
GA-EM	62.17 × 10^−4^	111.5	0.5
CDI-ver-EM	50.30 × 10^−4^	137.8	0.6
CDI-EM	26.18 × 10^−4^	264.8	1.2
GA-CDI-ver-EM	7.38 × 10^−4^	938.7	4.3
GA-CDI-EM	20.70 × 10^−4^	334.9	1.5
NHSP-ver-EM	110.02 × 10^−4^	63.0	0.3
NHSP-EM	108.99 × 10^−4^	63.6	0.3
CA-EM	116.11 × 10^−4^	59.7	0.3
EGNHS-EM	25.46 × 10^−4^	272.2	1.2
PS-EM	15.85 × 10^−4^	437.3	2.0
CU_control	145.31 × 10^−4^	47.7	-
CA-CU	3.88 × 10^−4^	1787.4	37.5
EGNHS-CU	62.39 × 10^−4^	111.1	2.3
PS-CU	11.54 × 10^−4^	600.8	12.6

* rate constant for enzyme thermal inactivation; **^†^** half-life time; **^#^** half-life time ratio calculated as t_1/2S_/t_1/2C_, where t_1/2C_ is a half-life time for a non-modified enzyme, whereas t_1/2S_ is for a modified one.

## Data Availability

The original contributions presented in this study are included in the article/Supplementary Materials. Further inquiries can be directed to the corresponding author.

## Supplementary Materials

The following supporting information can be downloaded at https://www.mdpi.com/article/10.3390/biom15040531/s1. Table S1: Effect of inhibitors on the activities of the *C. unicolor* C-139 and *E. meliloti* L3.8 laccases; Table S2: The predicted values of pK_a_, percent of SASA, reactivity, and salt bridges for lysine residues in 3D structure laccase from *E. melitoli* L3.8; Table S3: The predicted values of pKa, percent of SASA, reactivity, and salt bridges for lysine residues in 3D structure laccase from *C. unicolor* C-139; Table S4: Summarizing of POPS server calculation for hydrophobicity and hydrophilicity of SASA for 3D structural models of laccase from *E. meliloti* L3.8 and *C. unicolor* C-139; Table S5: Distance between selected lysine residue atoms on the surface of the 3D structure of *E. melitoli* L3.8 laccase; Table S6: Distance between selected lysine residue atoms on the surface of the 3D structure of *C. unicolor* C-139 laccase; Figure S1: The structural alignment between 3D structure models of laccases from *E. melitoli* L3.8 and *C. unicolor* C-139. Alignment was performed using a ChimeraX UCSF 1.9. The color barre indicates a root mean square deviation value for Cα atoms in Å (RMSD); Figure S2: Three-dimensional structural model of *E. melitoli* L3.8 laccase with annotated distances between Cα and ζN atoms of lysine residues located on the surface of the molecule; Figure S3: Three-dimensional structural model of *C. unicolor* C-139 laccase with annotated distances between Cα and ζN atoms of lysine residues located on the surface of the molecule.

## Appendix A

**Table A1:** Description of modifications.

Sample Shortcut (Name)	Description of Modifications
*Ensifer meliloti* laccase	
EM_control	Control experiment for untreated *E. meliloti* laccase
EGNHS-EM	EGNHS-modified *E. meliloti* laccase
CA-EM	CA-modified *E. meliloti* laccase
GA-EM	GA-modified *E. meliloti* laccase
CDI-EM	CDI-modified *E. meliloti*
GA-CDI-EM	GA-CDI-modified *E. meliloti* laccase
GA-ver-EM	GA-modified *E. meliloti* laccase having inactivated enzyme catalytic site with veratric acid
CDI-ver-EM	CDI-modified *E. meliloti* laccase having inactivated enzyme catalytic site with veratric acid
GA-CDI-ver-EM	GA/CDI-modified *E. meliloti* laccase having inactivated enzyme catalytic site with veratric acid
NHSP-EM	NHSP-modified *E. meliloti* laccase
NHSP-ver-EM	NHSP-modified *E. meliloti* laccase having inactivated enzyme catalytic site with veratric acid
Glc-EM	Glucose-modified *E. meliloti* laccase
Gal-EM	Galactose-modified *E. meliloti* laccase
Cel-EM	Cellobiose-modified *E. meliloti* laccase
Lac-EM	Lactose-modified *E. meliloti* laccase
Glc-ver-EM	Glucose-modified *E. meliloti* laccase having inactivated enzyme catalytic site with veratric acid
Gal-ver-EM	Galactose-modified *E. meliloti* laccase having inactivated enzyme catalytic site with veratric acid
Cel-ver-EM	Cellobiose-modified *E. meliloti* laccase having inactivated enzyme catalytic site with veratric acid
Lac-ver-EM	Lactose-modified *E. meliloti* laccase having inactivated enzyme catalytic site with veratric acid
PS-EM	Polymeric sucrose-modified *E. meliloti* laccase
*Cerrena unicolor* laccase	
CU_control	Control experiment for untreated *C. unicolor* laccase
EGNHS-CU	EGNHS-modified *C. unicolor* laccase
CA-SM	CA-modified *C. unicolor* laccase
PS-CU	Polymeric sucrose-modified *C. unicolor* laccase
