# Supplementary material for: Altering the Properties of Laccases from Ensifer meliloti (Sinorhizobium meliloti) and Cerrena unicolor by Chemical Modifications of Proteins
Source: Biomolecules. 2025 Apr 4;15(4):531. doi: 10.3390/biom15040531 (PMC12025185; doi:10.3390/biom15040531)
Supplement: Supplementary file 1 [file biomolecules-15-00531-s001.zip › Table S1-S6 and Figure S1-S3.pdf]

Altering properties of laccases from *Ensifer meliloti (Sinorhizobium meliloti)* and *Cerrena unicolor* by chemical modifications of proteins

Anna Pawlik [1,*], Radosław Drozd [2] and Grzegorz Janusz [1]

Table S1. Effect of inhibitors on the activities of the *C. unicolor* C-139 and *E. meliloti* L3.8 laccases.

	**Modification**																							
c [mM]	EM_ control	Glc-ver-EM	Glc-EM	Gal-ver-EM	Gal-EM	Cel-ver-EM	Cel-EM	Lac-ver-EM	Lac-EM	CA-EM	EGNHS-EM	PS-EM	GA-ver-EM	GA-EM	CDI-ver-EM	CDI-EM	GA-CDI-ver-EM	GA-CDI-EM	NHSP-ver-EM	NHSP-EM	CU_ control	CA-CU	EGNHS-CU	PS-CU
**% of activity in NaF**																								
**0**	100	100	100	100	100	100	100	100	100	100	100	100	100	100	100	100	100	100	100	100	100	100	100	100
**0.1**	75.69	72.65	49.47	54.35	59.69	46.58	54.86	66.07	63.08	58.46	73.17	68.69	73.13	66.76	61.16	66.61	58.89	76.61	55.68	58.55	81.78	85.37	87.28	80.21
**1**	32.64	25.10	15.72	18.56	20.30	17.64	16.95	23.30	25.76	28.70	29.90	29.80	24.68	24.83	22.56	26.38	24.30	26.90	11.48	20.32	55.81	65.65	61.85	54.01
**5**	5.08	0.48	0.64	0.68	0.21	0.81	0.51	3.26	1.88	1.57	3.90	5.56	01.10	0.54	0.86	0.00	0.48	0.29	0.00	3.62	21.35	22.45	21.12	18.23
**10**	1.51	0.30	1.20	0.68	0.49	0.78	0.41	0.21	0.00	0.00	0.47	1.25	0.00	0.00	0.00	0.57	0.12	0.15	0.13	0.40	9.97	9.18	8.98	8.52
**% of activity in NaI**																								
**0**	100	100	100	100	100	100	100	100	100	100	100	100	100	100	100	100	100	100	100	100	100	100	100	100
**0.1**	97.08	97.64	112.64	97.94	96.85	94.61	99.20	92.10	99.58	96.26	99.16	106.99	89.11	100	104.66	97.23	98.55	99.60	94.30	96.04	98.80	97.28	99.28	96.71
**1**	95.91	96.92	117.68	102.81	89.56	90.32	86.61	90.12	98.28	91.35	99.16	109.91	84.93	100	105.97	82.35	90.35	99.77	94.30	97.76	95.22	89.12	99.41	86.89
**5**	90.96	95.72	106.24	92.50	93.31	97.18	94.32	82.22	90.36	83.99	87.11	112.90	91.69	100	113.68	76.85	89.19	99.53	90.01	91.55	94.54	87.41	99.41	84.93
**10**	89.16	87.73	103.79	82.55	84.47	80.26	87.49	80.96	78.49	85.12	88.26	108.06	89.69	95.00	104.59	78.04	73.39	99.28	88.87	88.12	93.23	91.50	90.59	81.49
**50**	72.89	33.13	40.03	35.95	32.55	33.28	37.92	35.48	34.00	26.08	62.11	59.68	53.82	60.00	63.57	25.56	29.97	54.22	36.06	31.80	51.39	57.48	52.35	47.13
**100**	27.71	7.23	8.26	7.18	6.27	6.20	6.73	7.15	6.81	5.96	18.52	16.67	19.93	22.50	20.51	06.11	6.57	6.94	06.12	6.25	19.92	14.97	16.47	15.22
**% of activity in NaCl**																								
**0**	100	100	100	100	100	100	100	100	100	100	100	100	100	100	100	100	100	100	100	100	100	100	100	100

**0.1**	100	92.79	82.37	98.67	96.60	88.26	96.23	96.07	98.12	96.90	98.99	98.75	95.23	99.35	96.90	97.60	92.51	97.89	92.72	96.38	91.58	95.45	98.45	99.90
**1**	96.43	83.80	78.64	76.79	94.84	88.05	90.49	94.14	94.61	94.40	97.86	97.57	94.73	92.26	88.69	95.50	90.09	96.80	92.72	91.28	88.12	89.16	95.85	98.04
**5**	85.12	84.25	64.25	75.03	76.50	87.88	78.46	86.29	67.79	84.59	93.30	89.32	88.22	96.99	86.67	85.47	86.81	87.51	89.41	90.52	70.70	81.47	74.61	77.40
**10**	84.80	72.28	62.65	67.29	76.87	77.71	79.34	74.97	75.40	77.06	76.24	86.41	82.07	92.26	83.26	80.46	83.84	76.59	79.28	74.23	58.24	76.92	67.88	69.94
**50**	64.88	40.14	36.58	39.80	44.67	45.28	46.19	55.75	45.03	56.26	60.31	60.19	57.45	66.24	66.44	47.31	55.84	50.05	46.87	46.60	25.64	31.82	27.46	28.09
**100**	44.05	21.03	19.19	24.28	24.77	23.42	20.64	28.84	28.11	26.01	36.41	35.44	30.78	37.85	35.06	25.11	32.40	26.15	20.13	20.46	12.26	16.08	10.13	10.34
**% of activity in SDS**																								
**0**	100	100	100	100	100	100	100	100	100	100	100	100	100	100	100	100	100	100	100	100	100	100	100	100
**0.1**	75.78	61.65	73.02	92.74	86.80	92.59	86.17	84.61	58.07	77.13	98.81	90.37	94.99	68.68	88.33	59.09	77.35	73.91	88.03	68.52	86.37	71.76	73.99	87.21
**1**	65.63	55.63	65.33	57.88	75.84	73.33	28.40	65.66	46.98	12.61	94.56	86.18	63.47	73.89	78.70	57.24	44.43	72.12	88.03	45.86	62.68	67.94	44.51	68.60
**5**	27.73	0.00	0.00	0.19	0.00	8.63	5.97	0.61	7.97	0.00	28.51	27.14	0.00	0.00	0.40	0.00	01.10	0.20	0.00	0.00	33.66	21.18	18.99	30.20
**10**	15.47	0.00	0.00	0.00	0.00	0.00	0.25	0.64	0.00	0.00	6.12	16.51	0.00	0.00	0.00	0.00	0.00	0.00	0.00	0.00	17.02	16.26	11.54	18.95
**% of activity in DTT**																								
**0**	100	100	100	100	100	100	100	100	100	100	100	100	100	100	100	100	100	100	100	100	100	100	100	100
**0.1**	0.04	0.00	0.84	0.00	0.44	0.94	0.15	0.33	0.36	0.33	0.00	0.31	0.00	0.00	0.00	0.39	0.00	0.25	0.00	0.00	0.00	0.06	0.31	0.04
**1**	0.00	0.00	0.59	0.00	0.48	0.38	0.15	0.22	0.33	0.33	0.00	0.06	0.00	0.00	0.00	0.35	0.00	0.22	0.00	0.00	0.00	0.05	0.31	0.00
**5**	0.00	0.00	0.00	0.00	0.14	0.00	0.00	0.00	0.28	0.00	0.00	0.00	0.00	0.00	0.00	0.00	0.00	0.11	0.00	0.00	0.00	0.00	0.00	0.00

Table S2. The predicted values of pKa, % of SASA, reactivity and salt bridges for lysine residues in 3D structure laccase from *E. melitoli* L3.8.

Lysine number	pKa	% of SASA*	LysRe pH 7.4	LysRe pH 8.0	LysRe pH 9.0	Salt-Bridges
20	10.46	48.1	0.001148	0.004571	0.045709	
24	10.48	31.2	0.0003467	0.00138	0.013804	GLU221-LYS24
212	11.16	21.73	0.0004897	0.00195	0.019498	ASP218-LYS212
245	11.36	51.48	9.77237×10^{-5}	0.000389	0.00389	ASP235-LYS245
309	10.5	16.9	0.000302	0.001202	0.012023	ASP376-LYS309
333	11.17	61.97	0.00021	0.000832	0.008318	ASP328-LYS333
438	11.25	50.79	0.001281	0.005129	0.051286	ASP496-LYS488
444	10.49	40.28	0.000115	0.000457	0.004571	ASP421-LYS444
476	10.57	73.87	9.12011×10^{-5}	0.000363	0.003631	
488	10.32	44.59	0.000126	0.000501	0.005012	
529	10.36	48.31	0.001047	0.004169	0.041687	
555	11.03	30.32	0.000132	0.000525	0.005248	ASP537-LYS555
559	8.92	7.97	0.1	0.398107	3.981072	ASP344-LYS559
565	11.0	8.43	0.0021878	0.00871	0.087096	ASP548-LYS565

$$^{*}\text{SASA (\%)} = 100 \text{ x } \frac{\text{Total solvent accessible surface area aminoacid residue in protein 3D structure}}{\text{Total solvent accessible surface area of isolated aminoacid residue}}$$

Table S3. The predicted values of pKa, % of SASA, reactivity and salt bridges for lysine residues in 3D structure laccase from *C. unicolor* C-139.

Lysine number	pKa	% of SASA*	LysRe			Salt-Bridges
			pH			
			7.4	8.0	9.0	
39	10.53	24.71	9.33×10^{-6}	3.72×10^{-5}	0.000372	ASP100-LYS39<br>ASP127-LYS39
44	10.36	32.1	0.0001	0.000513	0.005129	ASP95-LYS44
58	11.52	22.53	0.0001	0.000398	0.003981	ASP487-LYS58
70	10.6	32.83	0.002138	0.008511	0.085114	
133	10.49	42.47	7.24×10^{-5}	0.000288	0.002884	ASP134-LYS133
275	11.0	44.43	4.46×10^{-5}	0.000178	0.001778	GLU190-LYS275
291	10.38	69.92	0.00085	0.003388	0.033884	
329	10.31	46.15	0.00095	0.003802	0.038019	
371	10.63	37.68	0.00054	0.002138	0.02138	
464	10.31	57.31	0.00112	0.004467	0.044668	
488	11.04	31.97	5.49×10^{-5}	0.000219	0.002188	ASP485-LYS488

$$^{*}\text{SASA (\%)} = 100 \text{ x } \frac{\text{Total solvent accessible surface area aminoacid residue in protein 3D structure}}{\text{Total solvent accessible surface area of isolated aminoacid residue}}$$

Table S4. Summarizing of POPS server calculation for hydrophobicity and hydrophilicity of SASA for 3D structural models of laccase from *E.meliloti* L3.8 and *C.unicolor* C-139

**Laccase source**	**Hydrophobic SASA Å²**	**Hydrophilic SASA Å²**	**Total SASA Å²**	**% of Hydrophobic SASA#**	**% of Hydrophilic SASA**	**R***
E. meliloti L3.8	16,789.74	13,950.33	30,740.07	54.6	36.4	1.20
C. unicolor C-139	12,121.44	8,997.31	21,118.75	57.4	32.6	1.34

#The % of hydrophobic and hydrophylic SASA was calualted according to formula; ((Hydrophobic or Hydrophilic SASA Å^2)/ Total SASA Å^2) x 100%
*The ratio between hydrophobic and hydrophilic SASA

The analysis of distances between lysine residues on the molecular surface was conducted for the compared laccases. Measurements were performed using UCSF ChimeraX 1.9, assessing the distances between the C*α* and *ζ*N atoms of lysine residues. Pairs of residues with distances not exceeding 18 Å were listed in Tables S5 and S6.

Table S5. The distance between selected lysine residues atoms on the surface of the 3D structure of *E. melitoli* L3.8 laccase.

Lysin residue		Distance Å	
		C*α*	*ζ*N
LYS	20<->24	7.0	12.2
LYS	24<->212	17.8	11.4
LYS	309<->333	9.8	16.3
LYS	438<->444	9.9	12.8
LYS	476<->488	14.2	23.9
LYS	529<->555	5.9	7.8

Table S6. The distance between selected lysine residues atoms on the surface of the 3D structure of *C. unicolor* C-139 laccase.

Lysin residue		Distance Å	
		Cα	ζN
LYS	39<->133	13.3	14.2
LYS	39<->70	11.2	15.0
LYS	44<->70	16.2	17.1
LYS	58<->488	6.7	11.8
LYS	70<->371	15.6	21.3
LYS	329<->488	19.9	16.5

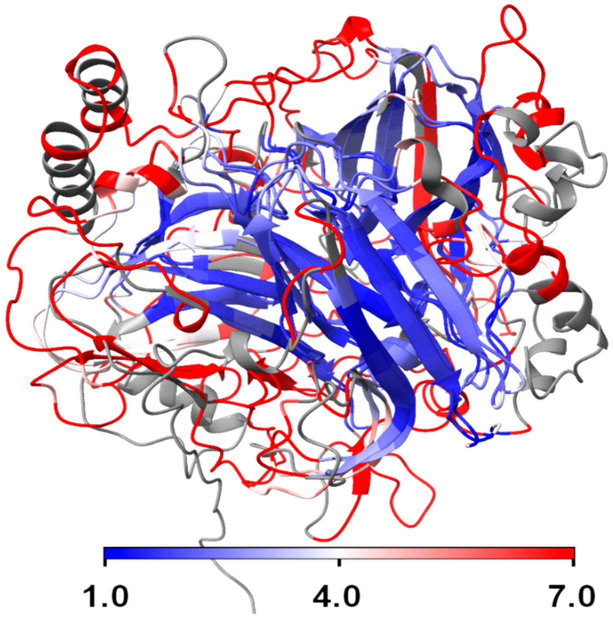

Figure S1. The structural aligment between a 3D structure models of laccases from *E.melitoli* L3.8 and *C. unicolor* C-139. Aligment was performed using a ChimeraX UCSF 1.9. The color barre indicate a root mean square deviation value for Cα in Å (RMSD).

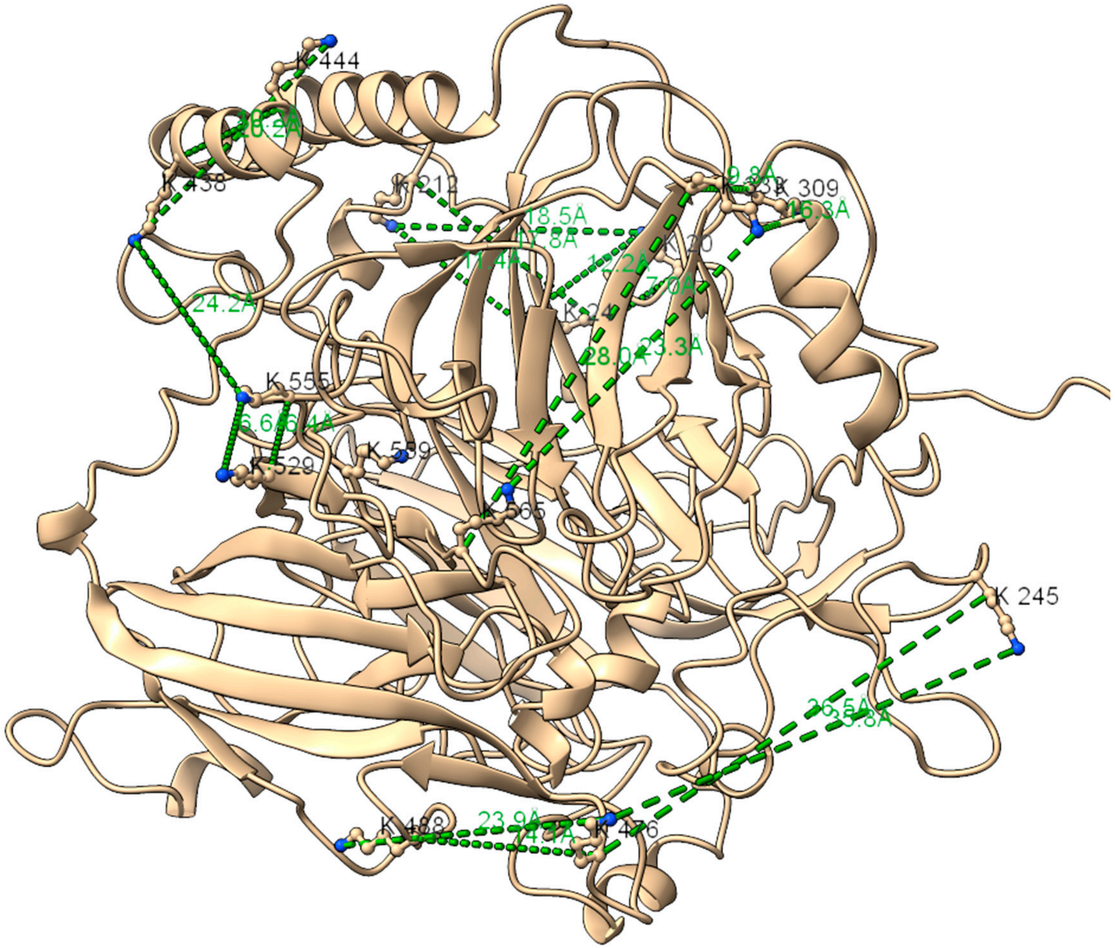

Figure S2. Three-dimensional structural model of *E. melitoli* L3.8 laccase with annotated distances between Cα and ζN atoms of lysine residues located on the surface of the molecule.

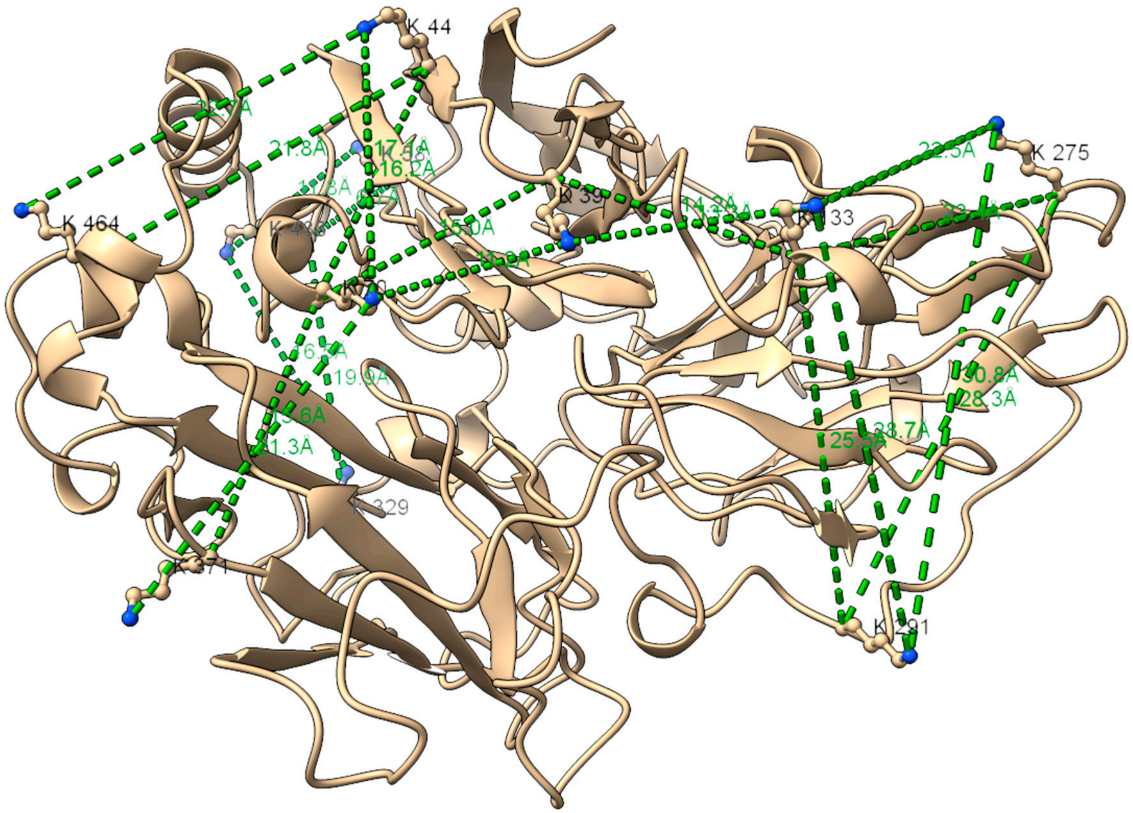

Figure S3. Three-dimensional structural model of *C. unicolor* C-139 laccase with annotated distances between Cα and ζN atoms of lysine residues located on the surface of the molecule.
